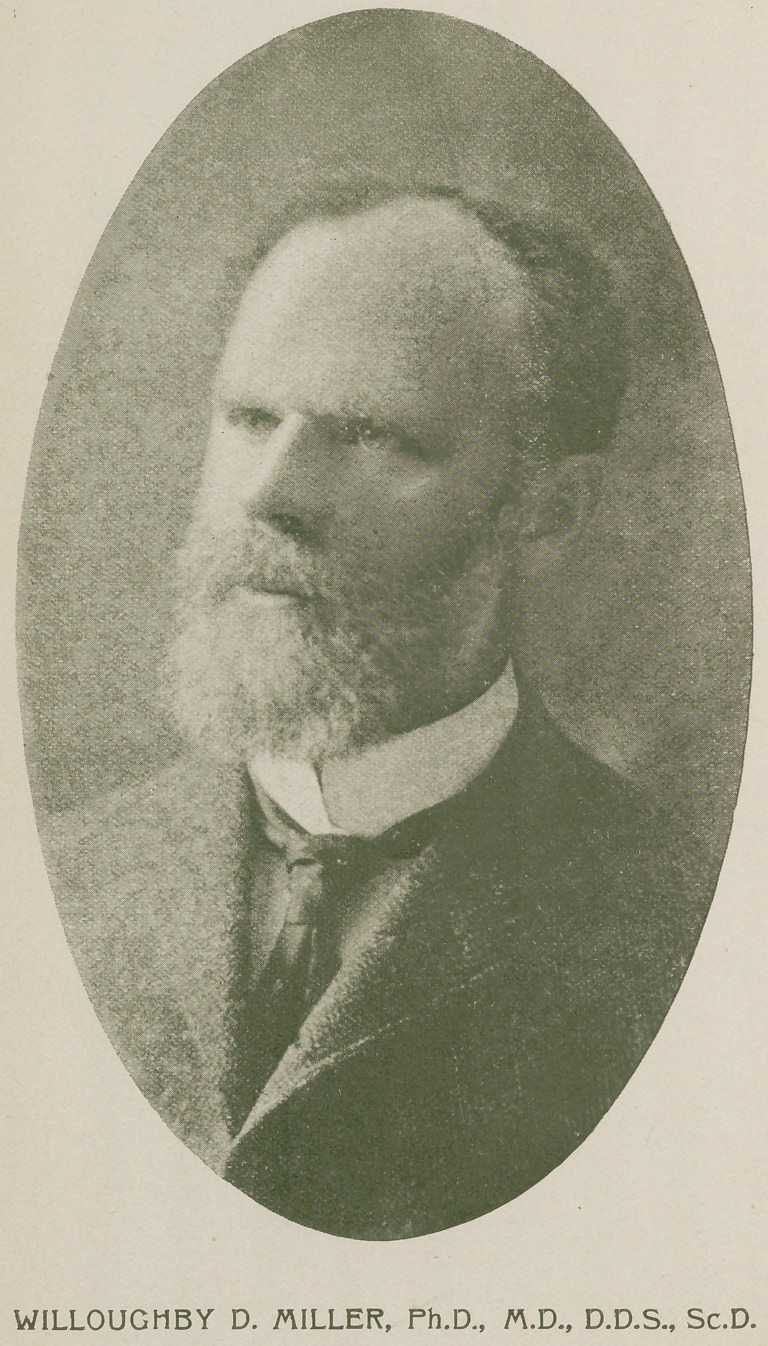# Death of Professor Willoughby Dayton Miller

**Published:** 1907-08-15

**Authors:** 


THE
### DENTAL REGISTER.

Vol. LXI. August 15, 1907. No. 8.

### EVENT AND COMMENT.

### Death of Professor Willoughby Dayton Miller.

At half past one o'clock, P. M., Saturday, July 27th, 1907, Professor W. D. Miller died in the City Hospital at Newark, Ohio, where five days previously he had undergone an operation for appendicitis.

The news of this sudden removal from a sphere of great activity, and of devotion to his beloved profession, will be a great shock to his many friends and admirers and bring a sense of incalculable loss to the dental profession throughout the world, as he unquestionably was the foremost scientist in his profession, and was just entering upon a new career of great importance to the profession of this country.

W. D. Miller was born August 1, 1853, near Alexandria, Licking County, Ohio, where his parents lived on a farm, which is still in the possession of the family and where he always spent his vacations when in this country. When he was twelve years of age his parents removed to Newark, Ohio, about ten miles distant from the farm, and he entered the public schools of that city and prepared for college. Graduating from the high school in 1871, he entered the University of Michigan that fall and took his degree of Bachelor of Arts in June 1875. While in college he devoted himself especially to mathematics and physics. After graduating he decided to adopt as his profession that of mathematical physics, and went to Scotland and entered upon a course of study in the Edinburgh University under Sir

William Thomson. He spent one year there, and then went to Berlin, Germany, for further preparation. Here, through overwork and study, his health gave away and he was compelled to stop his labors to recuperate, and it was during this enforced period of rest that he found his life's great work in a most unexpected way.

At this time Doctor F. P. Abbot was the representative American dentist at Berlin. He had married the daughter of a former American minister to Switzerland, Mr. Theo. S. Fay, and his home became well known to all Americans sojourning in the German Capital. Dr. Miller was a frequent guest at the home of Dr. Abbot and became acquainted with his older daughter whom he afterwards married. Through his intimate acquaintance with Dr. Abbot, Dr. Miller became interested in the chemical aspects of the dental art and made some researches for the Doctor concerning tin and gold fillings. These so greatly pleased Dr. Abbot that he induced Dr. Miller to return to America and take the dental course. This he did, graduating from the University of Pennsylvania in 1879. He returned to Berlin and took up the practice of dentistry and began the study of bacteriology with the famous Professor Koch.

From this time on he entered into the scientific work of his profession with all the zeal and enthusiasm which were so characteristic of the man, and which made him in due time the foremost dental scientist of the world. He continued a devoted student and at the same time carried on a large practice. He soon began to give the results of his scientific researches to the profession and thus entered upon a public career that involved a great demand upon his time and physical resources, so much so that he was once compelled to stop all work for a period of two or three years, and give his time to recuperating his health.

In 1884 he was made Professor of Dentistry in the University of Berlin, the highest honor that could be conferred upon him. It is said a German Professorship had never before this time been conferred upon a foreigner. In passing it is interesting to note that this act of the German government made Dr. Miller a German citizen, although he never took out German papers of citizenship, and consequently when he resigned his professorship in the fall of 1906 to accept a professorship in the University of Michigan he lost his German citizenship and became an American citizen again; or what is really the better way to put it, the German Empire lost an official subject.

In 1881 Dr. Miller began contributing his work through the dental societies, his first paper of importance was read that year at the annual meeting of the American Dental Society of Europe, at Wiesbaden. This was soon followed by a series of contributions to *The Independent Practitioner*, a dental journal edited by Dr. W. C. Barrett, of Buffalo, N. Y. These papers were upon the subject of the etiology of dental caries, and were received with much adverse criticism since they propounded a new theory as to the cause of dental caries. They resulted, as we know, in the establishment of the present theory as to the cause of this disease which is now universally accepted. The work done on this subject at once established Dr. Miller's reputation as a scientist, as it was thorough and faultless. From this time on he has been a constant contributor to the literary and scientific, and to a considerable degree, to the technical work of his profession.

In 1887 Dr. Miller took the "Rigorosum" examination for the medical degree in Berlin University. This is the most exhaustive of all examinations in this university, and he passed it with the predicate of "*Magna cum Laude*," and a record of fourteen out of a possible fifteen points, the next highest record obtained by anyone at that examination being eight. As a result of this examination, Dr. Miller's position in the Berlin University and in the minds of the German dentists was made secure, as the government had been severely criticised before for retaining on the faculty a foreigner, and many attempts were made to have the Minister of Education replace him with some German dentist. At the time of his leaving Germany, all prejudice had disappeared and the German dentists did all in their power to persuade him to remain and complete his work in Germany. It is reported that there was no one man to whom the German dentists were more loyal than to Dr. Miller, and American dentists practicing in Germany strongly opposed his return to this country, as he stood between them and the efforts continually being made to discredit their legal standing by German practitioners. His wise counsel and known integrity as well as his kind and generous disposition made him the most influential member of his profession in Europe. He was not only honored for his work and attainments, but he was universally beloved for his personal character, and there will now be many sad hearts where he was best known. Dr. Miller has had a great number of honors conferred upon him. He was an honorary member of 37 different professional organizations; the University of Michigan conferred upon him the honorary degree of Doctor of Philosophy; the University of Pennsylvania the degree of Doctor of Science; the Fourth International Dental Congress at St. Louis, in 1904, bestowed upon him a gold medal for a paper presented there entitled, "A Study of Certain Questions Relating to the Pathology of the Teeth." He had many other honors of which we are not sufficiently informed to speak. At the time when he was considering the question of leaving Berlin foı Ann Arbor, the German Emperor conferred upon him the very honorable title of "Kaiserlicher Geheimer Medizinalrath," which means privy medical councillor to the Emperor.

The faculty of the University of Michigan has for many years cherished the hope that some day it should have the help of Professor Miller in carrying out its plans of making Ann Arbor one of the best places of dental learning in the world, and when three years ago in a private conversation the writer learned that he was desirous of returning to this country that he might complete some work here for his profession that he very much wanted to do and which he could not well do in Germany, steps were immediately taken to bring him back to this country and to the University of Michigan. Professor Miller came to Ann Arbor and conferred with the dental faculty and University authorities and finding that the work required of him was so heartily in accord with that which he wished to undertake, and that the University was so willing to accord him the facilities for accomplishing it, he was persuaded to severe his relations with Berlin, in spite of the great pressure made in Germany to dissuade him. Friends, colleagues, dental societies and the highest government officials by their appeals made it difficult for him to leave the place he had so long and acceptably filled for a new and unknown field. As was characteristic of the man, although a rich merchant of Berlin offered to build, equip and support for him a complete dental research laboratory, if he would stay in Berlin, he decided to come to Ann Arbor because he could there do the work that he believed most needed to be done. Perhaps nothing can be said that would better illustrate the unselfish devotion of the man to his profession than to say that the work he planned to do here was of such a nature that it would probably not have added a greater honor to those already conferred upon him, but it would have contributed immensely to the future welfare of his profession, and this he considered much more important than any personal emolument or renown.

The idea that inspired Dr. Miller in recent years was to find some way by which the decay and loss of the teeth might be prevented one that could be universally applied and which especially should be within the means of people who are unable to pay for the present surgical methods of treatment. With this in view the last three years of his life were spent on an exhaustive research of the present mouth toilet preparations and prophylactic methods. He had but just begun this work and it was his purpose to continue this research until he could determine whether there was any chance of finding a solution of this problem in that direction. So far his work has thrown much light on the injuries resulting from the misuse of the tooth brush and injurious abrasive tooth powders, and has created a new interest in the chemical causes of wasting of the tooth structure. It was Dr. Miller's plan to continue this research and he had great hope that he would succeed in adding something of value to preventive methods, or at least that oral hygiene should be more scientifically practiced by the mass of the people than it now is.

There has been much comment both in this country and in Europe on Dr. Miller's decision to leave the land where so much of his life has been spent and where he had done the work that has brought to him so many honors. It is commonly supposed that it was his great love for his own country, and the natural desire that seems to come to all men to get back to the home of their kinsfolk and their places of birth that led him, in spite of the many strong inducements to remain, to sever his relations with Berlin and come to Ann Arbor, where the work and environment would be new and problematic. But Dr. Miller was a wise man and he looked into the future farther than most of us. He realized that there is a great work for the dental profession to do in the near future, and that so far as he could see there was no adequate preparation being made to meet the demand which is to be made for men who are competent to take up this work and do it scientifically. He saw that at Ann Arbor the means and material could be had for the development of scientific workers such as few other places offered. When called to accept the office of Dean of the Dental Department of the University of Michigan he made his acceptance contingent on a promise that he should be provided with suitable facilities for developing this line of work. This the Michigan authorities gladly agreed to do and the new building is now under way. It was his idea that he could select from the students passing through the school such men as he might be able to influence, and prepare for scientific study of the various dental problems.

He was greatly interested in the advancement of the dental educational standards and he hoped to be able to do much for the cause of higher standards in this country through his connec tion with the University of Michigan. He believed that the dental standards should be as high as the medical, and had well defined plans as to how this could be accomplished. He believed in the education of dentists as such and had little sympathy with the idea of a complete medical education as a foundation for the practice of dentistry. At the same time he strongly favored maintaining the instruction in the medical sciences so far as they had a value in equipping dentists for their practice, and for this reason he wanted the entrance requirements for a dental course brought up to those of medicine.

His last work was an address which he prepared for the annual meeting of the International Dental Federation, of which he was President. In this address he sets forth his views of this important question.

This brief statement of the life and work of this great man will in some measure help us to estimate the tremendous loss we have sustained in his death, at a time when he could have done so much for his profession and humanity. If his life shall stimulate others to take up the work with more earnestness we shall long have the benefit of his influence in making our profession of great usefulness to the world.

Those who knew Dr. Miller intimately speak fervently of his goodness of heart. He was naturally a modest man, but a man of very strong affection and was devoted to his family. He was an active supporter of the American church in Berlin, and gave his time and resources to it in the same lavish manner with which he devoted himself to his scientific work. He lived a true Christian life, and up to the last he gave evidence of the Christian faith and a firm belief in the life hereafter. He was a strong man in every way, and his loss to the world at this time is great and lamentable.

Dr. Miller leaves a widow and three children, the oldest, a son, is a medical practitioner in Berlin; a daughter, whose husband is W. G. Cady, is professor of physics in Wesleyan University at Middletown, Conn. and a younger daughter about thirteen years of age. Mrs. Miller will probably make her home with her daughter in Middletown, Conn.

## Figures and Tables

**Figure f1:**